# The obedient mind and the volitional brain: A neural basis for preserved sense of agency and sense of responsibility under coercion

**DOI:** 10.1371/journal.pone.0258884

**Published:** 2021-10-28

**Authors:** Emilie A. Caspar, Frederike Beyer, Axel Cleeremans, Patrick Haggard

**Affiliations:** 1 Consciousness, Cognition and Computation Group (CO3), Center for Research in Cognition & Neurosciences (CRCN), ULB Neuroscience Institute (UNI), Université libre de Bruxelles (ULB), London, United Kingdom; 2 Social & Moral Brain Lab, Department of Experimental Psychology, Ghent University, Ghent, Belgium; 3 School of Biological and Behavioural Sciences, Queen Mary University of London, London, United Kingdom; 4 Institute of Cognitive Neuroscience, University College London (UCL), London, United Kingdom; RWTH Aachen, GERMANY

## Abstract

Milgram’s classical studies famously suggested a widespread willingness to obey authority, even to the point of inflicting harm. Important situational factors supporting obedience, such as proximity with the victim, have been established. Relatively little work has focused on how coercion affects individual cognition, or on identifying the cognitive factors that underlie inter-individual differences in the tendency to yield to coercion. Here, we used fMRI to investigate the neural systems associated with changes in volitional processes associated with sense of agency and sense of responsibility under coercion. Participants either freely chose, or were instructed by the experimenter, to give mildly painful electric shocks to another participant, or to refrain from doing so. We have previously shown that coercion reduces temporal binding, which has been proposed as an implicit proxy measure of sense of agency. We tested how reduced agency under coercion related to differences in neural activity between free choice and coercion. In contrast to previous studies and to participants performing the task outside the MRI scanner, on average there was no effect of coercion on agency for participants in the scanner. However, greater activity in the medial frontal gyrus was reliably associated with greater agency under coercion. A similar association was found using explicit responsibility ratings. Our findings suggest that medial frontal processes, perhaps related to volition during action planning and execution, may help to preserve a sense of accountability under coercion. Further, participants who administered more shocks under free choice showed reduced activity during free choice trials in brain areas associated with social cognition. Possibly, this might reflect participants cognitively distancing themselves from the recipient of the shocks under free choice, whereas this was not observed under coercion.

## Introduction

Experiments conducted by Stanley Milgram [1, 2] showed that obeying orders can lead to extreme antisocial behaviours. Under certain circumstances, a majority of individuals could be coerced into inflicting apparent harm to others at levels generally deemed unacceptable (in fact, the ‘victim’ in Milgram’s studies was a confederate, who pretended to be harmed). While these studies have described the conditions under which coercion is effective, they have failed to address the central question of *how* coercion influences moral behaviour.

In everyday life, there are many -less dramatic- instances of following orders. Within reason, children are expected to obey their parents, students their teachers, workers their managers etc. We have previously shown that being coerced to perform a socially relevant action reduces temporal binding between actions and their outcomes in volunteer participants [3, 4]. Temporal binding has been linked to sense of agency (see [5, 6] for reviews), which is a central aspect of voluntary action, and is tightly linked to responsibility [7]. The sense of agency has been defined as the feeling that we are the authors (and thus potentially responsible) of our own actions and their consequences in the external world [8]. The reduction in sense of agency under coercion becomes highly relevant in cases of legal responsibility. For example, consider a worker who is instructed, or indeed coerced, by their manager to neglect safety protocols. If the worker experiences a reduced sense of agency under coercion, they may make riskier decisions than if they had felt a stronger sense of agency. Thus, sense of agency may be relevant to action control. However, most systems of law assume that healthy adults are responsible for the consequences of their own actions by default. Defences based purely on the *subjective* experience of voluntary control, or lack thereof, remain problematic and controversial [9].

Interestingly, Milgram reported that some individuals do resist the social constraint of receiving orders [10], but the neuro-cognitive mechanisms associated with this variability in ‘coercibility’ are not yet known. The present study explored such inter-individual variability in the neural processing of action under coercion. Specifically, we were interested in how coercion changes action-processing at the neural level, as a function of whether a person’s sense of agency and sense of responsibility are reduced by coercion, or not. To this end, we used functional magnetic resonance imaging (fMRI), while participants performed a coercion task adapted from previous work [3].

Sense of agency can be measured implicitly using time perception. In the intentional binding paradigm [11], participants estimate the delay between their action (i.e. a keypress) and an outcome (i.e. a tone). The perceived delay between a voluntary action and a tone is less than the perceived delay between a control event (such as an involuntary movement) and the same tone. The difference between these estimates is a measure of the intentional binding effect [12]. It has been previously shown that temporal binding can also be observed in the absence of motor actions, for example when participants observe actions in virtual environments, a phenomenon that was referred to as vicarious agency [13]. This suggests that visual information that allows for outcome prediction can itself generate temporal binding [14], and that intentional action is not necessary. Therefore, any study wishing to draw conclusions specific to voluntary action, or similar theoretical constructs, needs to be based on comparison of carefully matched conditions that should ideally differ in just one critical aspect.

Several factors can influence the perceived interval between actions and outcomes. For example, Borhani et al. [15], identified stronger binding for active than for passive movements, and stronger binding for actions involving an element of free selection, than for instructed actions. These were termed *motor binding* and *selection binding* respectively. The type of binding is defined by the contrast of interest in the experimental design. In the task previously used by Caspar et al. [3], two participants could deliver mildly painful electric shocks to each other in order to earn small monetary rewards. They performed this task either freely, or following orders of the experimenter. Results showed that participants’ temporal binding was reduced under coercion despite the fact that they were actually carrying out the action. The effect of interest recalls the ‘selection binding’ discussed by [15] and may be referred to as the ‘coercion effect on binding’ [3]. Crucially, this setup matched experimental conditions even beyond the visual observation control condition proposed by some other studies, as participants performed a motor action both in free choice and coercion trials. Conditions were thus matched in terms of both voluntary movement, and in terms of outcome predictability, and differed only in whether the precise action made was chosen by the participant, or specified by the experimenter. The only difference between our experimental conditions was thus volition *per se*, as all the motor, proprioceptive, predictive, and causal aspects were identical between experimental conditions.

Sense of agency can also be measured with explicit methods [5], by asking participants about their perceived control over actions or outcomes. Several studies have sought to understand to extent to which implicit and explicit measures of agency correlate [16]. While some studies reported no correlation between explicit measures of sense of agency and temporal binding [12, 17], other studies found significant positive correlations between temporal binding and explicit judgements of agency [18, 19]. It’s important to note that [17] used explicit judgments of authorship related to causality (i.e. ‘Did you cause this event?’), which differ from judgements of controllability [18, 20] or responsibility [21, 22]. Thus, someone can be well aware that they caused an effect, but nevertheless feel a low sense of control, for example when trying to ride an unfamiliar bicycle. It’s plausible that judgements of control or responsibility are correlated with temporal binding, whereas judgements of authorship are not. In the current study, we used temporal binding as an implicit measure of agency, in combination with explicit responsibility ratings.

Previous studies have shown that temporal binding is associated with increased activity in the supplementary motor area (SMA) [23], while explicit judgements of agency are negatively associated with activity in angular gyrus (AG) [24–27]. Previous studies also showed that free choices (compared to instructed choices) were associated with activation of brain areas associated with motor planning [28], such as the pre-supplementary motor area (preSMA), the right dorsal prefrontal cortex and the left intraparietal cortex. However, these studies focused on arbitrary choices between responses that did not produce meaningful outcomes. Thus, it is not clear whether similar cognitive processes underlie the action choices in situations where the agent’s action has valenced social consequences, as in coercion. One recent study found a negative relation between precuneus activity and explicitly measured sense of agency in a complex decision making task, involving social risk-taking [24]. Thus, if temporal binding is modulated by context- and task-relevant neural activity in the same way that explicit sense of agency is, one might expect that those frontal, parietal and temporal brain regions commonly associated with moral reasoning [29] and with social cognition [30], should also be correlated with coercion effects on temporal binding. We thus predicted that if the neural basis of basis of temporal binding can be influenced by the social context and outcome valence, we may observe that temporal binding correlates with activity in brain regions more frequently associated with social cognition. To evaluate how prosociality influences temporal binding and its neural basis, we also investigated how the number of shocks administered freely might be related to the subjective perception of being coerced neural processing of coercion.

## Methods

### Participants

Sixty participants (mostly University students) were recruited in same-gender dyads (i.e. female-female or male-male). None of the participants reported to know each other. To estimate the sample size, we used the effect size of the main effect of Condition (free-choice, coercive) of Experiment 2 in [3] of dz = 0.630. To achieve a power of .80, the estimated sample size was 22 dyads [31]. We set up the MRI sample size at 30 dyads in order to ensure a sufficient sample for analysis of the binding effect based on possible exclusion of participants. Within each dyad, each participant performed both the ‘agent’ role, and the ‘victim’ role. The *f*MRI data was collected only from the agents, and only those who performed the agent role first–this avoided possible order effects, as the participant performing the agent role after being the victim can be influenced by the number of shocks they received. Data of five participants were excluded, due to technical failure during the scan (N = 2), falling asleep (N = 2) and systematically disobeying instructions in the last run (N = 1). For the remaining 25 participants (14 females) who were scanned, the mean age was 23.0 years old (SD = 3.7). For the 25 participants who were not in the scanner (11 females), the mean age was 24.4 (SD = 5.2). Participants were recruited between December 2017 and March 2018. Inclusion criteria were being in good health, having no MRI counter indications, and being below 30 years of age. Participants were recruited from student populations at University College London and the local community. Participants were given a written study information sheet and provided written consent. The study was approved by the local ethics committee of the University College London (0847/009).

### Materials and procedure

**Fig 1:**
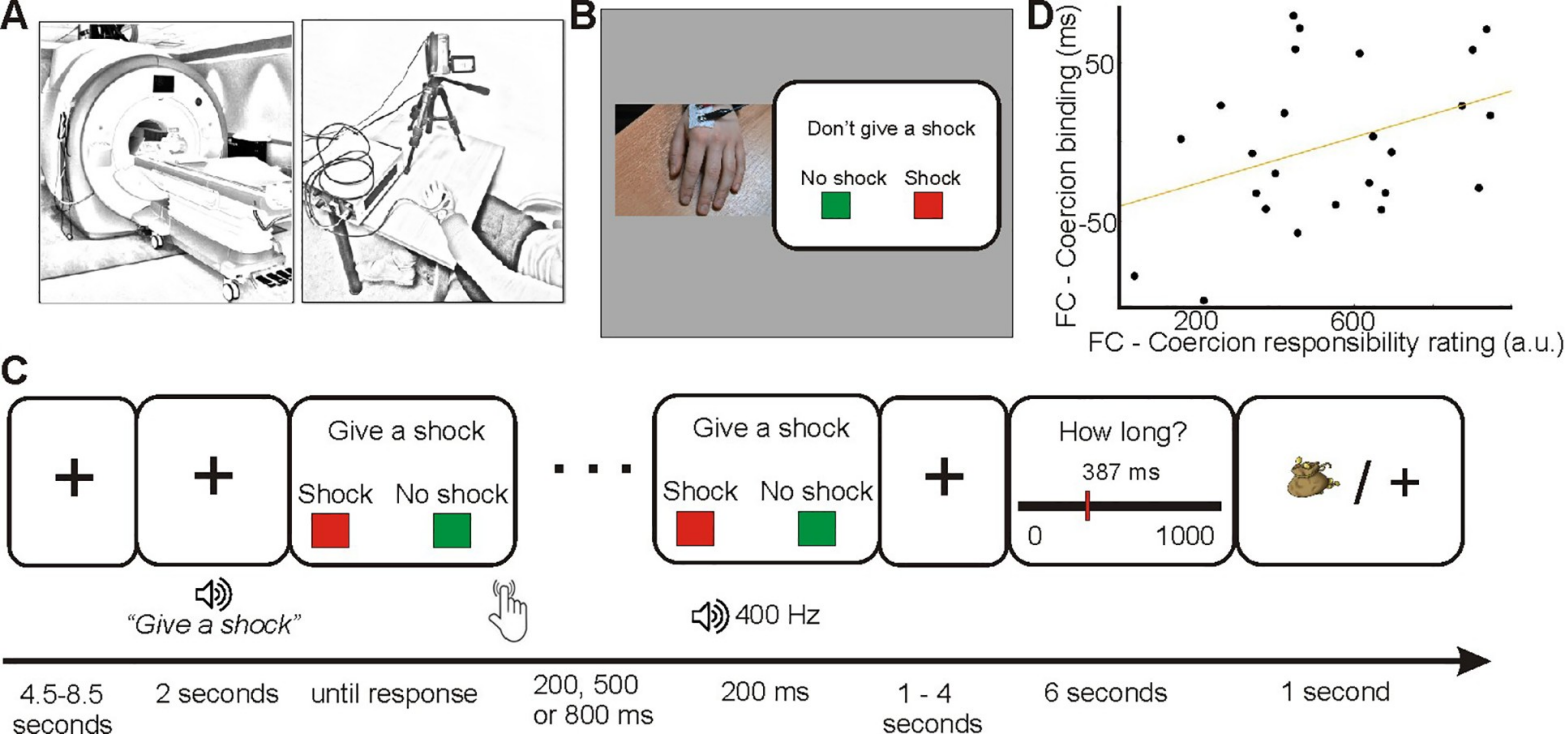
Task setup. **A** shows the physical setup; one participant (the agent) performed the task inside the MRI, the second (the ‘victim’) in an adjacent room, with a visual ink via camera. **B** shows the visual presentation inside the MRI: on the left, a live camera feed of the recipient’s hand was shown; on the right, task stimuli were presented. **C** shows the temporal outline of a single trial. **D** shows the correlation between the coercion effect on binding and the coercion effect on explicit control ratings.

Two pilot experiments were conducted on two different samples than the one used in our main paradigm in order to set up the following task parameters (see S1 File): Upon arrival in the laboratory, both participants received instructions and provided informed consent. They then underwent a procedure to determine their individual pain threshold for the electrical stimulation, as described previously [3] and in the (S1 File). Electrical stimulation was calibrated to be mildly painful. The participant who was assigned to be the agent first was then brought into the scanner while the participants who was assigned to be the ‘victim’ sat in the room adjacent to the scanner with the experimenter and was connected to the machine delivering the painful stimulations (Fig 1A). The agent had two screens in front of her (Fig 1B). On one screen, a video of the recipient’s left hand was displayed in real time. On each trial, agents first received an auditory instruction (‘Give a shock’ / ‘Don’t give a shock’ in coercion trials; ‘You can decide’ in free choice trials), see Fig 1C. Unbeknownst to participants, these instructions were pre-recorded to allow precise timing. To increase the authenticity of the procedure, each sentence was recorded 4 times with small variations in the voice and displayed randomly. In addition, the audio recordings included a background sound similar to interphone communications.

After receiving the verbal instruction, a picture of two rectangles, a red one labelled ‘SHOCK’ and a green one labelled ‘NO SHOCK’, was displayed on the left and right bottom of the screen. The key-outcome mapping varied randomly on a trial-wise basis but the outcome was always fully congruent with the mapping seen by the participant. Agents could then press one of the two buttons. Pressing the SHOCK key delivered a shock to the victim while pressing the NO SHOCK key did not deliver any shocks. Both choices were available on each trial, regardless of any instructions given. A tone followed each button press after 200, 500 or 800ms. Action-tone intervals were shuffled with the constraint that within a set of six trials, each interval was presented twice. On trials where a shock occurred, it was delivered at the same time as the tone to avoid introducing a temporal bias in interval estimates. Participants were asked to estimate the interval between their button press and the tone (following the procedure described in [32]. An analogue scale with ‘0’ on the left side of the scale and ‘1,000’ on the right side was displayed on the screen. A red position marker was displayed on that scale with a number, corresponding to the marker’s current position in ms. The starting position of the marker varied randomly on a trial-wise basis. Participants had 4 buttons, two for each hand, to adjust the position of the marker by steps of 100 ms and 1 ms. After a fixed duration of 6 seconds, their answer was saved and the trial finished. Each shock sent to the ‘victim’ was rewarded of +£0.05 in the two experimental conditions. If participants selected to send a shock, a drawing of a money bag was presented for one second after they estimated the duration of the interval between their button press and the resulting tone.

Thus, the experimental conditions were identical in terms of visual, auditory and somatosensory events, and the only difference was the content of instructions (‘You can decide’ in the free-choice condition vs. ‘Give a shock / don’t give a shock’ in the coercive condition). Further, the instruction phase was separated from the action execution phase, because the response-outcome mapping was presented only after the instructions were given. Thus, decision making—whether or not to give a shock–was separated from motor preparation and execution.

The task was split into 8 blocks of 12 trials each, 4 blocks free choice and 4 blocks coercion (presented in 4 fMRI runs with two task blocks each). At the end of each task block, participants rated their explicit sense of responsibility (i.e., ‘How responsible did you feel for the outcomes of your action?’) over the outcomes of their actions, using a continuous scale ranging from ‘not responsible at all’ to ‘fully responsible’, with a cursor controlled by the left and right index finger keys on the button boxes. In coercion blocks, all participants were instructed to deliver a shock on 50% of trials and in the free-choice blocks, they were free to decide the number of shocks to administer. Throughout the experiment, the participant could observe the receiver’s hand through a video link, and could thus observe that electric shocks elicited a visible muscle twitch. After the task, anatomical images were recorded. Participants then performed the task outside the scanner, and reversing the agent and ‘victim’ roles. The behavioural data for this second phase of the experiment were analysed, but there was no corresponding *f*MRI data. The second phase was included to ensure that the dyadic interaction was reciprocal.

### MRI data acquisition, preprocessing and analysis

MRI images were recorded using a 3.0-Tesla Siemens trio scanner and a 32-channel head coil. T1-weighted structural images were recorded with the following specifications: matrix = 240x256; 176 sagittal slices; voxel size = 1x1x1mm. Four runs of functional images were recorded (matrix = 64x64; 48 transversal slices in ascending order; 195 volumes per run; TR = 3.3 seconds; TE = 30ms; voxel size = 3x3x3mm^3^, including a .3mm slice gap). Processing of MRI data was carried out in SPM12 [33] with the toolbox extensions Marsbar [34] and rfxplot [35]. Images were slice timing corrected, realigned to the first image of the first run, normalised into MNI space and smoothed with an 8mm kernel.

At the first level, we defined separate regressors for free choice and coercion trials, starting with the presentation of the auditory instructions and lasting until the tone (= action outcome) was presented. Regressors of no interest included button presses, the agency rating scale, tone presentation and motion regressors.

At the first level, we defined the free choice > coercion contrast. At the second level, we used this contrast to investigate general effects of coercion in a one-sample t-test, as well as to set up between-subject regression analyses. We defined three regressors to investigate inter-subject variability in the neural processing of coercion: the coercion effect on binding; the coercion effect on responsibility ratings, and the number of shocks administered in free choice trials. Each participant’s mean binding score was obtained by subtracting the estimated action-tone interval for each trial from the actual action-tone interval. The difference between the mean binding for coercion trials and that for free-choice trials was taken as the coercion effect on binding. In three separate analyses, we tested for modulation of the free choice > coercion contrast by each regressor.

For all whole-brain analyses, we used a significance threshold of p < .05 FWE-corrected at the cluster level, with an initial threshold of p < .001 uncorrected.

## Results

### Behavioural results

We tested temporal binding scores and explicit responsibility ratings for normality using Kolmogorov-Smirnov tests. While the tests for binding-coercion (p = .055) and responsibility ratings–free choice (p = .051) approached significance, the difference scores free choice-coercion used in the following correlation analyses were normally distributed for both measures (p = .2).

#### Number of shocks freely delivered by the participants

Behavioural data for MRI and non-MRI participants are shown in Table 1. Due to the high variability in the number of shocks administered freely (see S1 File), we did not split up reaction times or judgment errors by shock/no-shock, as 10 participants showed fewer than 10 trials for one of these choice options. In previous studies, the factor shock/no-shock did not interact with the effects of interest [3, 4, 36].

**Table 1:** Summary of behavioural data.

	MRI Group		Behavioural Group	
	Mean	Min/Max	Mean	Min/Max
N shock trials free choice	25.82	1 / 46	21.1	3 / 48
N disobedience trials	1.4	0 / 10	2.0	0 / 12
	**Mean**	**Std**	**Mean**	**Std**
RT Free choice (s)	1.28	0.51	1.16	0.37
RT Coercion (s)	1.06	0.25	1.02	0.34
Binding Free choice (ms)	72.6	131.0	191.9	82.8
Binding Coercion (ms)	74.0	135.8	176.1	86.8
Responsibility Free Choice (a.u.)	854.9	124.5	915.26	122.8
Responsibility Coercion (a.u.)	315.9	241.6	348.6	288.0
Coercion effect on Binding (ms)	-1.4	49.1	15.8	41.7
Coercion effect on Responsibility (a.u.)	538.9	252.4	366.7	305.3
Coercion effect on RT (s)	0.22	.34	0.13	0.18

Unless otherwise mentioned, our analyses focused on the MRI sample only. In the coercive condition, participants were ordered to inflict 24 out of 48 shocks, randomly. One participant never followed the orders of the experimenter in one block, by systematically pressing on the other button related to the order received, and this participant was therefore excluded. In the free-choice condition, participants were told that they were entirely free to decide to deliver a shock or not to the other participant. On average, agents administered 25.82/48 (SD = 14.76, minimum: 1/48, maximum: 48/48) shocks on free choice trials. While this average is similar to the number of shocks given under coercion, the mean value masks large individual differences, with many participants showing either a higher or lower number of freely given shocks (S1). Thus, the proportion of shocks that participants freely chose to deliver could differ substantially from the 50% of shocks instructed under condition. Among MRI participants included in the analysis, disobedience in coercion trials was overall low (mean number of disobeyed trials = 1.4, SD = 2.4; min = 0; max = 10). Disobeyed trials were excluded from MRI analysis.

#### Coercion effect on binding

We calculated temporal binding scores for each subject, by subtracting each interval estimate from the actual response-tone interval for that trial. This difference represents judgement errors, with a high positive value showing a strong temporal binding effect (e.g. if the response-tone interval was 500ms, and the participant estimated it to be 400ms, this would yield a binding score of 100). We then averaged these scores separately for free-choice and coercion trials. On average, we did not observe a significant difference in binding between Free-Choice and Coercion trials (M_free_ = 72.6ms, SD_free_ = 131.0 ms; M_coercion_ = 74.0ms, SD_coercion_ = 135.8 ms; one-sided t-test, *p* = .557) for participants performing the task inside the MRI scanner. In contrast, participants performing the task outside the scanner showed significantly stronger binding in free choice compared to coercion trials (M_free_ = 191.9ms, SD_free_ = 82.8 ms; M_coercion_ = 176.1ms, SD_coercion_ = 86.8 ms; one-sided t-test, t_24_ = 1.9; *p* = .036; Cohen’s-d = .18). Importantly, the absolute estimation error (i.e. the absolute value of the difference between estimated and actual intervals) was comparable between groups, as was within-subject variability in interval estimation errors (mean absolute estimation error = 222.6 ms inside scanner/ 227.6 ms outside scanner, SD = 68.6 ms / 63.9 ms, t_48_ = -.3, *p* = .792; mean standard deviation = 154.8 ms/ 158.2 ms, SD = 41.0 ms / 31.8 ms, t_48_ = -.3, *p* = .741). Thus, group differences in temporal binding and its sensitivity to coercion could not be explained by generally impaired interval estimation performance in the MRI environment. Rather, there was unusually high inter-subject variability in coercion effects on binding, suggesting that the MRI environment (e.g. being in a different room than the experimenter) could have modulated the effects of coercion in some participants.

Participants inside the scanner did, however, show lower explicit responsibility ratings under coercion than free choice trials (M_free_ = 854.9(a.u.), SD_free_ = 124.5; M_coercion_ = 315.9(a.u.), SD_coercion_ = 241.6; *p* < .001, t_24_ = 10.7).

For each subject, we calculated the coercion effect on binding, by calculating the difference ‘binding under free-choice’–‘binding under coercion’. A strongly positive coercion effect corresponds to a decrease in temporal binding under coercion, and thus putatively to a strong reduction in sense of agency under coercion. We did the same for explicit responsibility ratings. We then correlated the coercion effect on participants’ responsibility ratings with the coercion effect on their binding scores. As implicit and explicit measures of agency could be positively correlated, we performed a one-sided test. This showed a modest association in the predicted direction (r = .38, *p* = .03). Participants who showed a stronger reduction in judgements of responsibility under coercion also showed stronger reductions in binding under coercion. Thus, coercion effects on binding in the scanner were generally lower than those outside the scanner, but between-subject variability in coercion effects on binding was related to coercion effects on subjective responsibility. This suggests coercion effects on binding may depend on the extent to which participants’ subjective experience was affected by coercion (rather than, for example general effects of being in a scanner environment on time estimation).

### MRI results

#### Main effect of coercion

**Fig 2:**
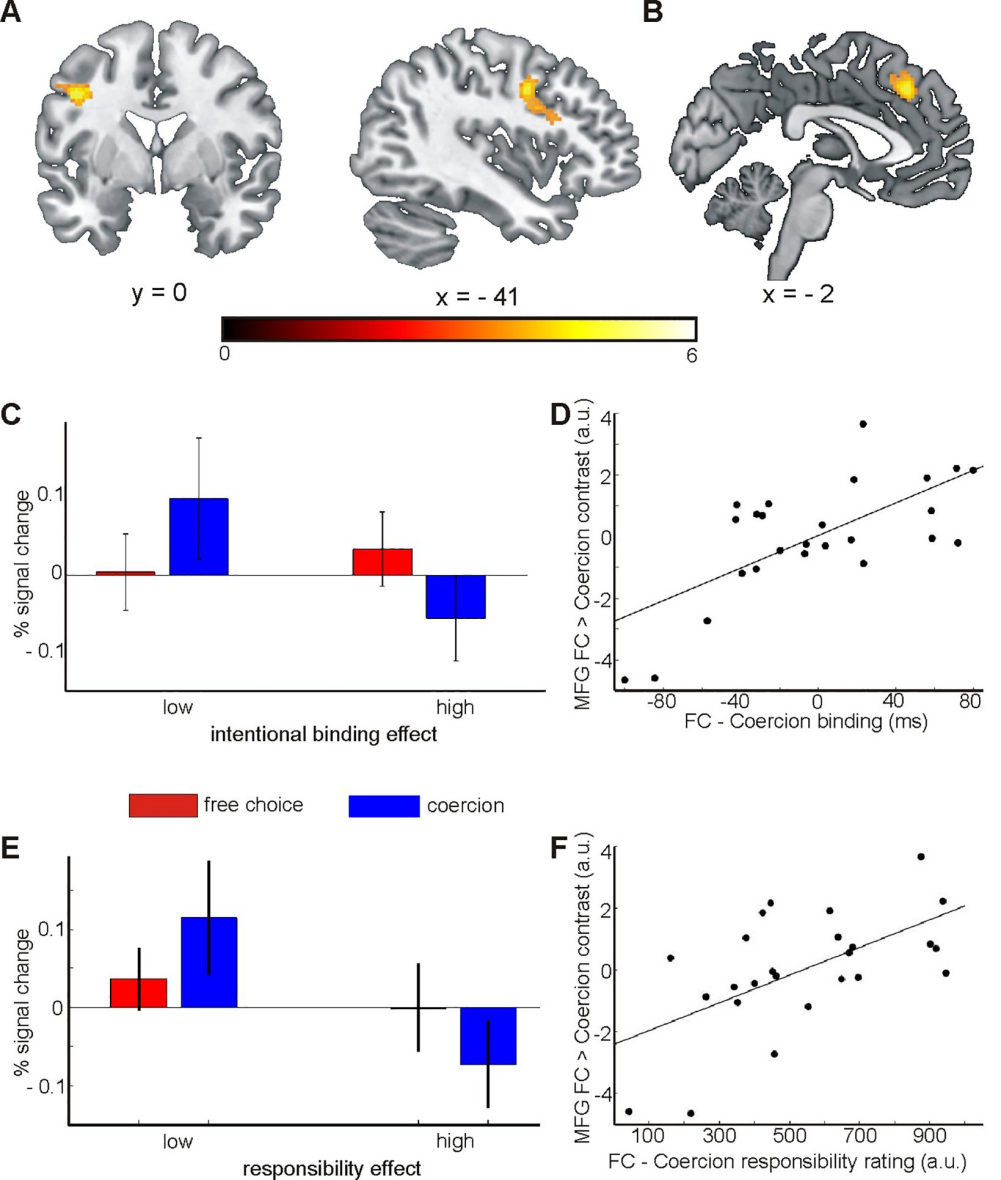
Main effect of coercion and interaction with coercion effect on binding. **A** shows the main contrast Coercion > FreeChoice. **B** shows the MFG cluster of the regression of the binding coercion effect against the FreeChoice > Coercion contrast. **C** shows percent signal change for the region shown in **B**, for participants sorted by median split according to their coercion effect on binding. **D** shows the correlation between the Free choice > Coercion contrast in the MFG and the Free Choice > Coercion difference in binding. **E** shows the percent signal change for the same region, with participants sorted by median split according to their coercion effect on explicit responsibility ratings. **F** shows the correlation between coercion effects on MFG activity and responsibility ratings. Error bars represent standard errors of the mean.

Contrasting Coercion > FreeChoice trials showed increased activity in the left inferior frontal gyrus (lIFG; Fig 2A; Table 2). The reverse contrast (FreeChoice > Coercion) showed no significant effects.

**Table 2:** Whole-brain fMRI analyses results.

Region	MNI coordinates (X,Y,Z)	cluster peak t-value	cluster size (N voxels)	Cluster-level p-value
***Coercion > Free choice***				
Inferior frontal gyrus (Left)	-44, 0, 38	4.79	424	.004
***Regression*: *coercion effect on binding***				
Medial frontal gyrus	0, 32, 42	5.17	247	.035
***Regression*: *number of shocks***				
Superior temporal gyrus (Left)	-64, -38, 4	7.15	1956	< .001
Thalamus (Right)	14, -22, 10	6.48	946	< .001
Precentral gyrus (Right)	56, -12, 42	5.8	594	< .001
Superior parietal lobule (Right)	26, -58, 44	4.61	296	.013
Posterior cingulate	-8, -36, 32	4.57	1030	< .001
Middle temporal gyrus (Right)	60, -8, -4	4.39	540	.001

Voxel-level significance threshold p < .001; cluster-level Family-Wise Error corrected at p < .05.

#### Modulation of the neural processing of coercion by reduced temporal binding

While the high variability in coercion effects on temporal binding mentioned above weakened the overall behavioural effect, we were able to use this variability to investigate the neural correlates of individual differences in the coercion effect on binding. For each subject, we subtracted the binding score for coercion trials from that for free choice trials, such that a higher score indicated a stronger reduction of binding under coercion. We then regressed this score against the FreeChoice > Coercion MRI contrast. This showed a positive effect for a cluster in the medial frontal gyrus (MFG; Fig 2B; Table 2) with a whole-brain approach: participants with more strongly reduced binding under coercion also showed relatively lower activity in the MFG under coercion, see Fig 2B. Thus, relatively stronger activity in this area under coercion was associated with a preserved sense of agency, as measured through the method of time perception.

The regression results are shown in Fig 2D, with the FreeChoice > Coercion contrast value in the MFG shown for a 5mm sphere centered on the cluster peak. Please note that this scatter plot is shown for visualisation purposes only and should not be understood as an effect size indication.

To further visualise the effects underlying this regression result, we performed a median split on participants based on the size of their individual coercion effect on binding. We then defined a 5mm sphere around the peak of the MFG cluster and extracted activity values for free choice and coercion trials. As can be seen in Fig 2C, there was a trend towards increased activity in this area under coercion for those participants whose binding was less strongly reduced under coercion, but not in participants whose binding was more strongly reduced under coercion. As before, this median split was performed for visualisation purposes only. No statistical analyses were performed on the post-split data.

Crucially, MFG activity was not correlated with the *quality* of the interval estimates, calculated as the slope of interval estimates against the actual intervals (r = .12, *p* = .58), and the coercion effect*MFG correlation held when controlling for inter-individual differences in slope (r_part_ = .69, *p* < .001).

#### Modulation of the neural processing of coercion by reduced sense of responsibility

We first performed an ROI-analysis to test whether reduced sense of explicit responsibility under coercion was similarly related to activity in this MFG cluster, as observed for interval estimates. To this end, for each subject, we calculated the mean responsibility rating for free choice blocks and subtracted from that the mean rating for coercion blocks. This gave us an individual score of the extent to which each subject reported a reduced subjective sense of responsibility under coercion. We used this score as a regressor against the FreeChoice > Coercion MRI contrast and performed an ROI analysis using the above-mentioned MFG cluster (5mm sphere around the peak activation). This showed a positive correlation between the responsibility effect and MFG contrast (t = 3.5, *p* < .001), see Fig 2F. Descriptively, participants with preserved explicit ratings of responsibility under coercion showed higher activity in this area under coercion, with participants who reported reduced responsibility under coercion showing the opposite effect (Fig 2E). This suggests a common neural basis of reduced temporal binding and reduced sense of responsibility under coercion. We therefore tested whether the shared correlation between MFG activity and coercion effects on binding on the one hand, and coercion effects on responsibility on the other could fully explained by the correlation between coercion effects on binding and on responsibility scores (see above). The partial correlation MFG*responsibility remained significant even when controlling for temporal binding (r_part_ = .49, *p* < .05) and the partial correlation MFG*binding remained significant even when controlling for responsibility (r_part_ = .61, *p* < .01). Note that these partial correlation coefficients should not be interpreted as effect size measures, because they are post hoc analyses on our fMRI findings. In addition to this ROI-based analysis, we also performed a whole-brain regression analysis on the coercion effect on responsibility ratings. This showed no additional significant effects.

#### Modulation of the neural processing of coercion by individual disposition to freely administer shocks

**Fig 3:**
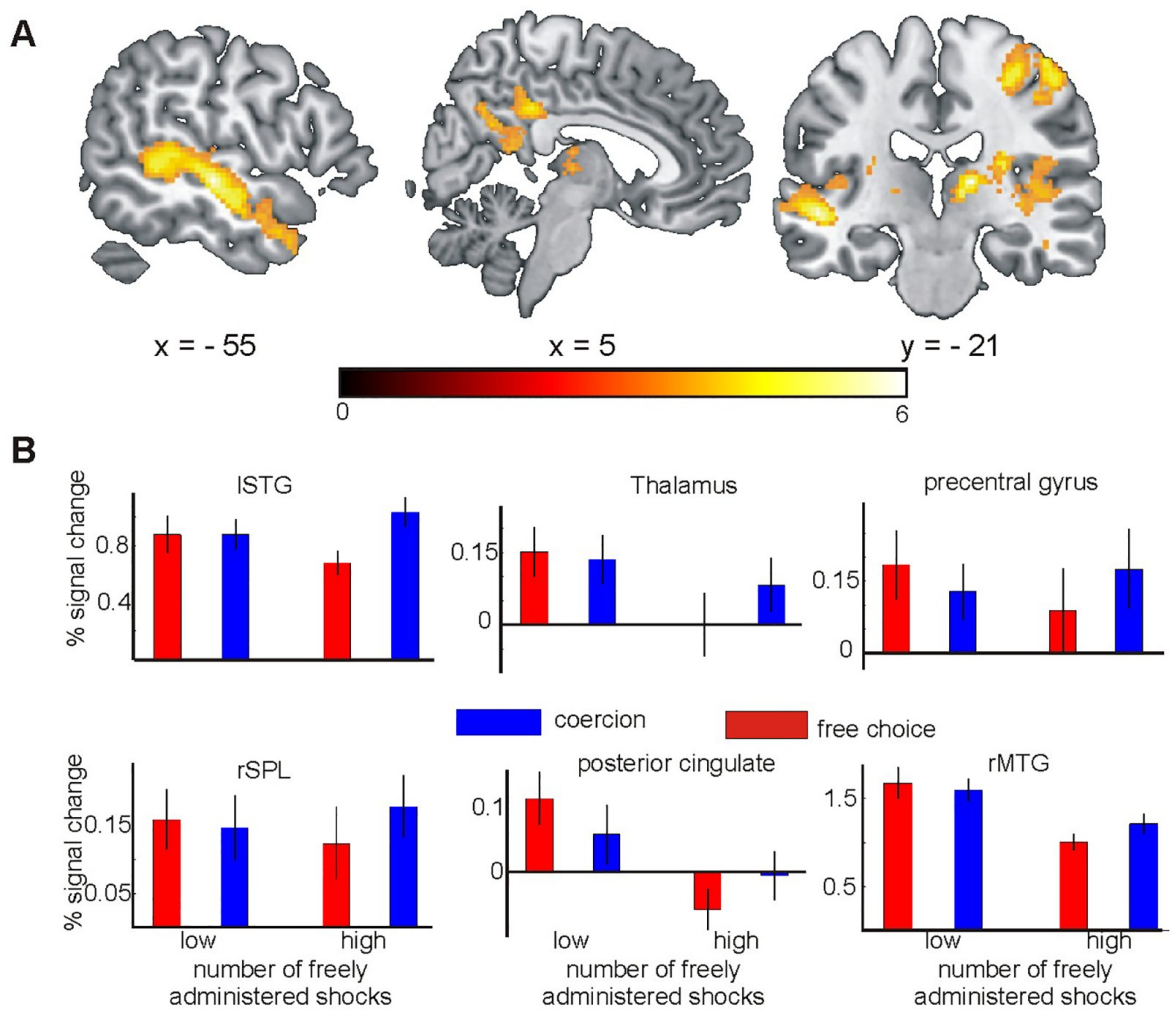
Interaction of freely administered shocks and coercion. Fig **A** shows the regression of number of freely administered shocks against the FreeChoice > Coercion contrast. For the shown regions, participants who delivered more shocks showed relatively less activity in free choice trials. **B** shows the median split for the regions shown in **A**.

Finally, to test whether the neural processing of coercion in this task context was affected by the individual disposition to freely administer shocks, we investigated the interaction between coercion and the number of shocks delivered in free choice trials. We used the number of shocks that participants delivered in free choice trials as a between-subject regressor against the FreeChoice > Coercion contrast. This showed a negative effect for the right superior parietal lobule (rSPL), posterior cingulate / precuneus, right middle temporal gyrus (MTG), left superior temporal gyrus (lSTG), right thalamus and right precentral gyrus (Fig 3A; Table 2). Thus, relatively stronger activity in these areas under free choice was associated with a lower number of shocks delivered. Post-hoc median split analyses for these areas revealed a general trend within free choice trials, namely that those participants who administered more shocks showed lower activity than individuals who delivered fewer shocks (Fig 3B). These results held when additionally including coercion effects on binding and responsibility ratings as additional regressors in the design (see figure in S1 File).

## Discussion

We investigated inter-individual variability in the neural processing of coercion, focusing on the roles of reduced temporal binding and responsibility under coercion. In a free-choice condition, participants were free to choose whether to give a mildly painful electric shock to another participant in exchange for money. In a coercion condition, the same participants were instructed by the experimenter either to give identical shocks, or not. We used the temporal binding effect as an implicit measure of sense of agency [12] alongside explicit ratings of responsibility.

In our study, where participants delivered electric shocks that they knew to be painful, yet tolerable and non-harmful, disobedience rates were low. However, inter-individual differences in responses to coercion are highly important in real-life circumstances [37, 38]. A preserved sense of agency under coercion, as measured by a low coercion effect on temporal binding, and a preserved sense of responsibility for action outcomes might conceivably function as a protective mechanism against problematic social and moral consequences of inappropriate obedience. Our study sought to identify the putative mechanisms in the human brain that might underlie these effects.

### The relationship between reduced agency and responsibility under coercion, and the neural processing of coerced actions

Several studies have investigated the effect of free and forced choices on the sense of agency [39–41], yet those studies used non-relevant, arbitrary action outcomes. In our task, in contrast, each action had social and monetary consequences that presumably felt relevant to the participant. In this context, we observed a different relationship between sense of agency and neural activity patterns than previous studies, the significance of which is discussed below.

Across subjects we found that participants who showed stronger reduction of temporal binding under coercion, also showed less medial frontal gyrus (MFG) activation in coercion trials. Put another way, participants who retained a high sense of agency–as measured by binding—under coercion showed increased activity in the MFG under coercion, compared to free choice trials. It should be noted that we refer to a *relative* reduction in binding under coercion, compared to free choice. As absolute binding scores are difficult to interpret, we cannot say whether participants with a greater coercion effect on binding showed reduced binding under coercion, or increased binding under free choice.

Similar correlation patterns were also found for explicit responsibility ratings, suggesting that the result is not merely a spurious correlation linked to a role of the MFG in time estimation. While intentional binding is often considered an implicit, or pre-reflective measure of sense of agency, responsibility ratings may be more strongly influenced by contextual factors, such as the awareness that one was carrying out orders, or moral considerations based on participants’ understanding of responsibility. It is thus not surprising that the correlation between these two measures in the MRI participants was modest. This finding is relevant regarding the ongoing discussion on the relationship between implicit and explicit measures of agency [16]. Here, we show that temporal binding can be positively correlated with responsibility ratings, and that both measurements are partially associated with the same underlying neural mechanism.

Notably, MFG activity was not globally increased in the free-choice condition in comparison with the coercive condition. Rather than representing a general neural correlate of action selection, MFG activity correlated with temporal binding and feeling of responsibility specifically under coercion. Our findings thus suggest that resilience against agency- and responsibility-reducing effects of coercion is associated with recruitment of volitional processes reflected in MFG activity. The cluster we found to correlate with both temporal binding and responsibility effects lies at the anterior border of the pre-SMA, in a region associated with voluntary action selection [42, 43].

One might postulate that participants with stronger volition under coercion show greater MFG activations for the simple reason that they themselves voluntarily intended to do anyway whatever the coercive instruction dictated. However, in our design, the coercive instruction to shock or not was *varied randomly*, so participants’ acting in accordance with a consistent voluntary preference for one outcome would make them subject to relevant coercion on at least some trials.

**Fig 4:**
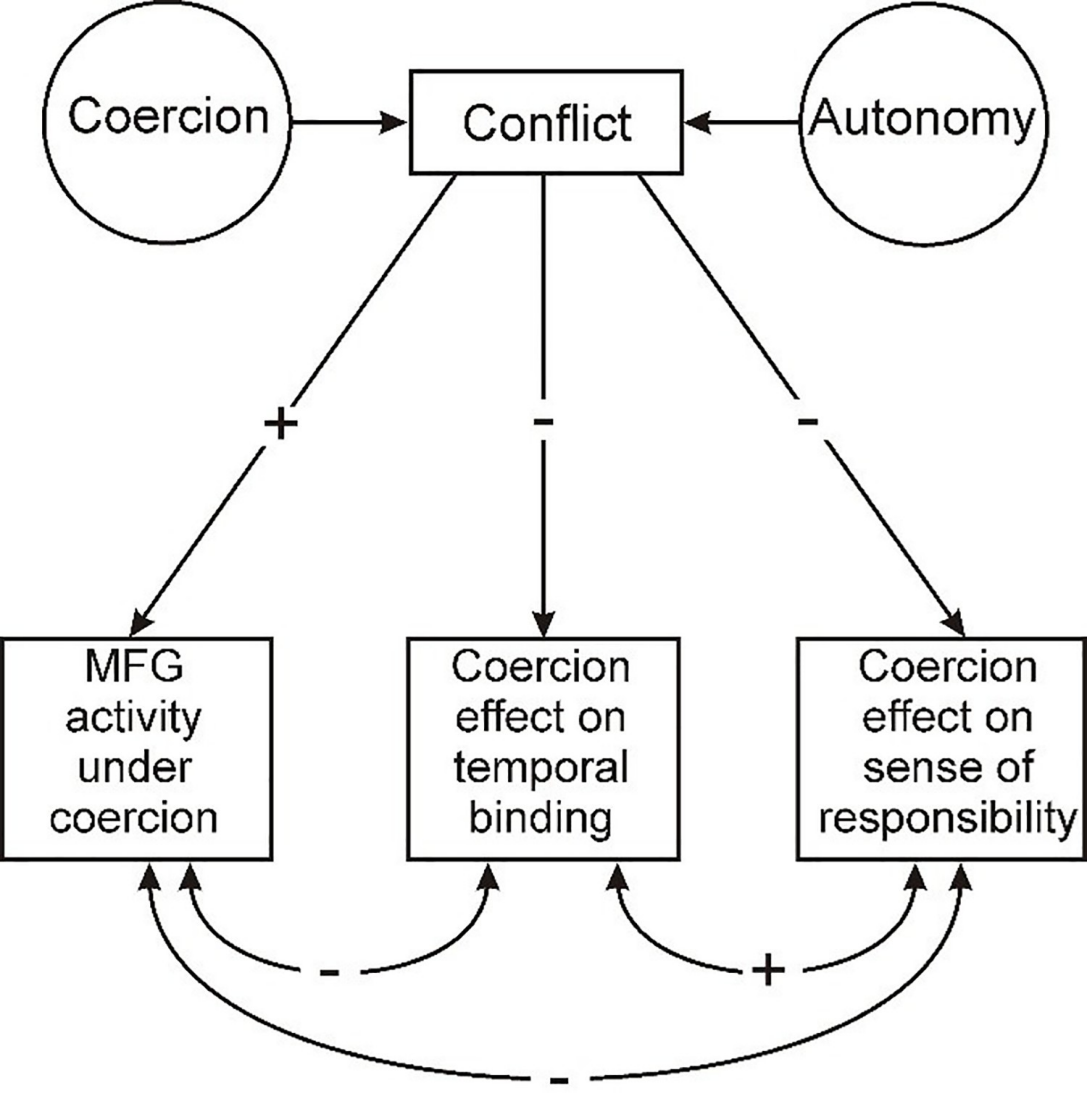
Schematic model showing the proposed underlying cognitive mechanism of the relationship between MFG activity, temporal binding and sense of responsibility under coercion.

Commonly, MFG is more strongly activated in free choices than in instructed choices for simple motor tasks [28, 44, 45]. However, the particular region of MFG activated in our study is involved in overcoming the conflict between two competing action plans [46]. It has also been found activated for another important aspect of voluntary action and free will, namely the intentional inhibition of planned actions [47]. Accordingly, we suggest that, for some participants, following a coercive instruction involved a strong source-level conflict with volitional action control, based on the individual’s efforts to act autonomously. A schematic representation of the concepts involved is shown in Fig 4.

The paradigm used here is not designed to elicit disobedience: participants are explicitly shown that the shocks they deliver are not harmful, though they are painful. For some participants the coercion condition contains a higher ratio of shocks than they administered under free choice, for others, the reverse is the case. The effect of coercion on temporal binding and responsibility ratings is observed independently of whether a shock is delivered or not. Indeed, the temporal binding task involved judging the interval between action and tone, so the occurrence or non-occurrence of a shock at the time of the tone was in fact incidental to the judgement task. Thus, we do not claim that coercion induces a strategic displacement of responsibility for harmful action outcomes. Rather, we show preserved temporal binding and sense of responsibility under coercion are associated with patterns of neural activation that suggest strong volitional control. Future studies should test this interpretation, perhaps by using direct behavioural measures of volition, rather than relying on neural activation patterns.

### Coercion and disposition to freely administer shocks

Participants who gave a high number of shocks in free choice trials showed relatively less activity on free choice trials in a range of brain areas, including temporal lobe areas, angular gyrus and posterior cingulum / precuneus, and subcortically in the thalamus. These cortical areas are traditional mentalizing areas, and the thalamus has also been found to be activated in some social cognition tasks [30]. A tentative interpretation of these findings is that, in free choice trials, participants who chose to deliver more shocks, may have been less cognitively engaged, and may have mentalized less about the consequences of their actions on their co-participant. Suppressed mentalizing during free choice trials may have made it easier for participants to deliver mildly painful stimuli to the other person. In contrast, under coercion, activity in these areas was similarly high for both groups of participants. This suggests that the potential to displace responsibility onto the person giving the orders might make it easier for participants to contemplate the consequences of their actions for others. Given that these findings are based on neural activity patterns alone, future studies should test these theories using behavioural measures of perspective taking. This finding is nonetheless consistent with a former MRI study, which showed that the number of shocks that agents freely chose to deliver was associated with the activity in the temporo-parietal junction (TPJ, [48]), a brain region associated with emotional perspective taking and theory of mind [49, 50].

### Main effect of free choice vs. coercion

In coercion trials, compared to free choice, we found increased activity in the left inferior frontal gyrus, reaching from Broca’s area into the precentral gyrus. Involvement of Broca’s area might reflect processing of the verbal instructions [51, 52]. In contrast to previous studies [28], we did not find increased SMA activity in free choice trials, or any other brain areas. However, the instructed choice conditions of previous studies typically instructed participants to perform arbitrary motor behaviours [53, 54], used speeded responses [28, 54], and mixed free vs. instructed trials. In contrast, in our task, free choices were not arbitrary, but meaningful and socially relevant. Further, because our free choice trials occurred in a separate block, and without any instructed trials being present, action selection could be done in advance, in the inter-trial interval. These design features may explain the absence of SMA and pre-SMA activations associated with our free choices. Another recent study reported that the left superior medial and mid orbital gyri in the vmPFC were more active while making a decision in the Free-choice condition compared to following orders in the Coercion condition [48]. The fact that we do not observe such difference in the present study may be the result of a reduced experience of coercion because the experimenter was not physically present in the MRI room, while in [48], they were.

### Behavioural effects

While coercion effects on binding were clearly related to neural processing of coercion on the between-subject level, coercion had no effect on binding at group level in those participants tested in the MRI scanner. Participants tested outside the MRI scanner did however show an effect in the expected direction [3], and in line with the results of the pilot experiments (S1 File). Importantly, the absolute error of interval estimations was not affected by the scanner environment. The unusual physical environment for MRI scanning, in which the agent is in a different room from both the experimenter who coerces them, and from the recipient whom they shock, could plausibly reduce the overall effect of coercion on sense of agency. Indeed, one variation of Milgram’s original study [10] involved the experimenter giving orders via a phone, without being physically present in the room with the participant. In this situation, participants were much more likely to disobey the experimenter’s coercive instructions, thus demonstrating a weakened effect of the coercive instructions. Studies of authoritarian politics note that coercion typically requires strong presence and signaling by the coercive power [55].

Further, overall temporal binding was reduced in the MRI scanner, compared to when participants performed the task at a standard computer outside the scanner. This effect may be due to the unusual setting. For example, the task in the MRI scanner was performed without vision of the hands, and using response boxes, while the task outside the MRI scanner was performed with vision of the hands and using a computer keyboard. These factors could influence the experience of controlling action.

Across participants, the strength of the coercion effect on binding had a modest association with the coercion effect on explicit responsibility ratings in the predicted direction, further supporting our interpretation, that the reduction of the coercion effect by the MRI environment uncovered meaningful inter-individual variability.

### Future directions

Our findings suggest two future research directions. First, most previous studies have focused on the effects of coercion when performing harmful actions. However, coercion in the context of positively-valenced actions is relatively common. For example, parents and educators may use a mild form of coercion to facilitate pro-social behaviour in children (e.g. insisting that a child apologise for harm they caused). Including positive action outcomes, as opposed to painful shocks, would allow for further testing of the role of opposition to coercion, and sense of agency, in preserved sense of responsibility, and might have important implications for possible pro-social uses of coercion.

Second, future research should investigate whether preserved agency and sense of responsibility under coercion are associated with an increased probability of disobeying coercive orders. While it would be ethically problematic to increase the (perceived) harm of actions, in order to obtain higher disobedience rates, future studies might potentially decrease the perceived costs of disobedience, for example by further removing perceived experimenter supervision.

## Conclusions

We have shown increased activity in medial frontal gyrus is related to a preserved temporal binding under coercion (which we interpreted as a preserved sense of agency), and also to a preserved sense of responsibility for action outcomes. This might reflect the process of overcoming the conflict between obeying authority and one’s preference for acting in accordance with one’s own free choice. Further, participants who delivered more mildly painful shocks to another participant under free choice, showed reduced activity in free choice trials in brain areas associated with social cognition. This might reflect cognitive distancing from the recipient of the shocks. Future studies should attempt to extend these findings by using behavioural measures of volition and social cognition.

## Supporting information

S1 File Supplemental information. Methods and results for pilot studies, supporting figures.
(DOCX)
