# Supplementary material for: The obedient mind and the volitional brain: A neural basis for preserved sense of agency and sense of responsibility under coercion
Source: PLoS One. 2021 Oct 28;16(10):e0258884. doi: 10.1371/journal.pone.0258884 (PMC8553174; doi:10.1371/journal.pone.0258884)
Supplement: S1 File — Methods and results for pilot studies, supporting figures. (DOCX) [file pone.0258884.s001.docx]

*S1 File*

**PILOT 1. METHOD**

***Participants***. Twenty female participants were recruited in dyads. During the recruitment procedure, we ensured that participants were not close friends or relatives, by mixing people studying different academic courses to create the dyads. Participants received between €20 and €26 for their participation. No participants were excluded based on the linear trend analysis for the three delays used and no participants withdrew their participation. The mean age was 23 years old (SD=2.53). The study was approved by the local ethical committee of the Université libre de Bruxelles (001/2017).

**Linear trend analysis.** For time intervals, to identify participants for whom the action-tone intervals did not gradually increase with action-tone intervals, we performed a linear trend analysis (LTA) with contrast coefficients -1, 0, 1 for the three delays we used, similarly to previous studies (Caspar et al., 2016). We used LTA statistical to verify that participants’ estimation of the time intervals were similar with real time intervals. LTA can be used to observe if the tendencies of the data follow a linear trend. To be included in the final dataset, this trend value hand to be both positive (i.e., perceived time increasing with actual interval duration) and statistically significant with p<0.05.

***Procedure***. On arrival at the experimental laboratory, participants read an information sheet about the experimental procedure and the aim of the experiment. After reading the document, the experimenter repeated the explanations orally, and participants were invited to ask any questions. Afterwards, the two co-participants signed their individual consent forms simultaneously, ensuring that they were each aware of the other’s consent.

The roles of the participants were assigned based on where participants happened to sit when they first arrived in the room. One participant started by being the agent and the other participant the ‘victim’. These roles were reversed mid-way through the experiment, making the procedure fully reciprocal, similarly to the method used by Caspar et al. (2016). The agent and ‘victim’ were seated at a table, facing each other. A keyboard was placed between them, oriented towards the agent but visible by both. The experimental task ran on a computer located on the agent’s right side, with the screen visible only to the agent and to the experimenter. The agent was instructed to press a key on the keyboard after receiving the experimenter’s instruction. The keyboard included two keys explicitly labelled: ‘SHOCK’ and ‘NO SHOCK’. Pressing the first one delivered a painful electric shock to the victim; pressing the second one delivered no shock. The agents earned €0.05 each time they pressed the ‘SHOCK’ key. They earned no extra money if they decided to press the ‘NO SHOCK’ key. There were two experimental conditions. In the coercive condition, the agent had to follow the experimenter’s instruction. In the free-choice condition, the agent was free to decide which key to press. To have a comparable outcome effect (shock vs. no shock) in the analysis, agents again received €0.05 each time they administered a shock in the coercive condition. The experimenter was sitting in another table, facing both the agent and the ‘victim’. A keyboard was placed in front of her, with three keys labelled ‘SHOCK’, ‘NO SHOCK’ or ‘DECIDE’. The experimenter gave a verbal instruction in both conditions. In the coercive condition, the experimenter said ‘*give a shock*’ or ‘*don’t give a shock*’ to the agent. In the free-choice condition, the experimenter told the agent ‘*you can decide*’. After having given the instruction orally, the experimenter pressed the key corresponding to her order. This makes the instruction also appearing on the agent’s screen. This procedure ensured that participants really knew that the person making decisions was the experimenter, and not someone who would have been only reading what would appear on the screen of the computer. A fixation crossed of 2s, 4s or 6s was introduced between the instruction given by the experimenter and the possibility for the participant to press a key (see FIGURE 1).

The experimental conditions were not entirely blocked such as in previous studies (Caspar et al., 2016; Caspar et al., 2017; Caspar et al., 2018) but divided in 4 smaller blocks of 24 trials each (12 trials per experimental condition). The order of the condition was randomized between blocks. The order of conditions was counterbalanced across dyads, but the order was always the same for the two co-participants belonging to the same dyad.

Shocks were delivered using a constant current stimulator (Digitimer DS7A) connected to two electrodes placed on the back of victims’ left hand, visible to the agent. Participants’ individual pain threshold was determined for the two participants after they had signed the consent form, before starting the experiment. This threshold was determined by increasing stimulation in steps of 1 mA, following the procedure described in Caspar et al. (2016). The mean stimulation level selected by this procedure was 34.7 mA (SD=16, pulse duration: 200 µs). This procedure ensured that both participants knew how painful the shocks were and were fully aware that shocks were real.

Whether the agent delivered a shock or not, a tone occurred after the keypress. The delay between key press and tone was set to vary randomly at 200, 500, and 800 ms. If a shock was delivered, it occurred at the same time as the tone. A visible muscular twitch on the victim’s hand was observable by the agent when the shock occurred. The participants’ task was to estimate the delay between the agent’s keypress and the tone. Participants were informed that the delay would vary randomly on a trial-by-trial basis, between 1 and 1,000 ms (they were reminded that 1,000 ms equals 1 s). Participants were also told (1) to make use of all possible numbers between 1 and 1,000, as appropriate, (2) to avoid restricting their answer space, and (3) to avoid rounding. An analogue scale with ‘0’ on the left side of the scale and ‘1,000’ on the right side was displayed on the agent’s screen (**Fig. S1**). A red rectangle was displayed on that scale with above a number, in ms, which corresponded to its position. The starting position of that rectangle varied randomly on a trial basis, so agents were additionally told not to be influenced by the starting position of that rectangle to give their answer. Agents had 2 buttons: if they pressed the right button it decreased the answer by steps of ‘-1’ and if they pressed the left key it increased the answers by steps of ‘+1’. Pressing the keys for a long time increased the speed of the steps, to reach the desired answer more rapidly. ‘Victims’ received a paper sheet with 48 empty boxes in which to write their time estimates in each condition of the task, which was hidden from the view of the agent to avoid any bias.

Brain activity was recorded using a 64-channels electrode cap with the ActiveTwo system (BioSemi) and data were analysed using Fieldtrip software. The activities from left and right mastoids and from horizontal and vertical eye movements were also recorded. Amplified voltages were sampled at 2048 Hz. Data were referenced to the average signal of the mastoids and filtered (low-pass at 50 Hz and high-pass at 0.01 Hz). Because of the EEG recordings, agents were further instructed to wait a minimum of 2 s in a relaxed position before pressing a key, so as to obtain a consistent and noise-free baseline. Participants were additionally instructed not to move for up to 1 s after the tone and asked to avoid blinking when they pressed a key.

In a post-session questionnaire, participants were invited to estimate in percent how much responsible they felt in each experimental condition. They were also invited to describe in a few words what they had felt during the experiment, and any reactions they had to the experiment. Finally, participants were paid separately based on the financial gain earned during the experiment.


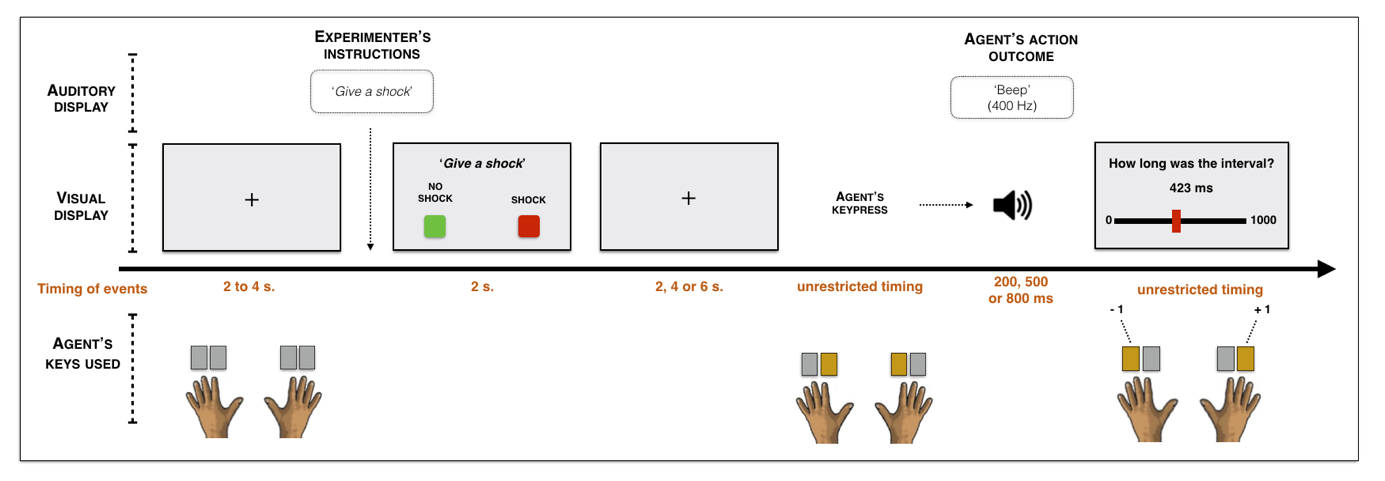


Fig. S1. Display of the agent’s task in pilot 1.

**PILOT 1. RESULTS**

***Interval estimates.*** We conducted a repeated-measures ANOVA with Condition (Free-choice, Coercive) and Shock (Shock, No shock) as within-subject factors on agent’s interval estimates. Three participants never inflicted shocks to the ‘victim’ in the free-choice condition and one inflicted shocks on every trial. These four participants were thus not taken into account in the ANOVA. We observed that, even if interval estimates were shorter in the free-choice (490 ms, SD=16.56) than in the coercive condition (517 ms, SD=19.66), the main effect of condition was not significant (*p*=.152). Neither the main effect of outcome (*p* > .2) nor the interaction (*p* > .1) were significant.

***Electrophysiological results.*** Given that the number of trials per condition was lower than in previous studies (48 vs 60, Caspar et al., 2016; 2017; 2018), we only analysed the auditory N1 according to the experimental condition, without taking into account whether a shock had been delivered or not. A paired sample t-test indicated that the amplitude of the auditory N1 was larger in the free-choice condition (-9.59 µv, SD=3.77) than in the coercive condition (-8.50 µv, SD=4.15, t(19)=-2.377, *p* = .031, Cohen’s d=.524), thus replicating the original study (Caspar et al., 2016).

**PILOT 2. METHOD**

***Participants***. Twenty female participants were recruited in dyads. During the recruitment procedure, we ensured that participants were not close friends or relatives, by mixing people studying different academic courses to create the dyads. Participants received between €20 and €26 for their participation. No participants were excluded based on the linear trend analysis for the three delays used. One participant withdrew her participation. On the remaining participants, the mean age was 22.78 years old (SD=2.22). The study was approved by the local ethical committee of the Université libre de Bruxelles (001/2017).

***Procedure***. The procedure of Pilot 2 was almost entirely similar to the procedure used in Pilot 1. The only difference is that we removed the fixation crossed of 2s, 4s or 6s that was present between the instruction given by the experimenter and the possibility for the participant to press a key (Fig. S2). By removing time restriction, we though to increase the feeling of freedom of agents even if they still had to wait the experimenter’s instruction to press a key.


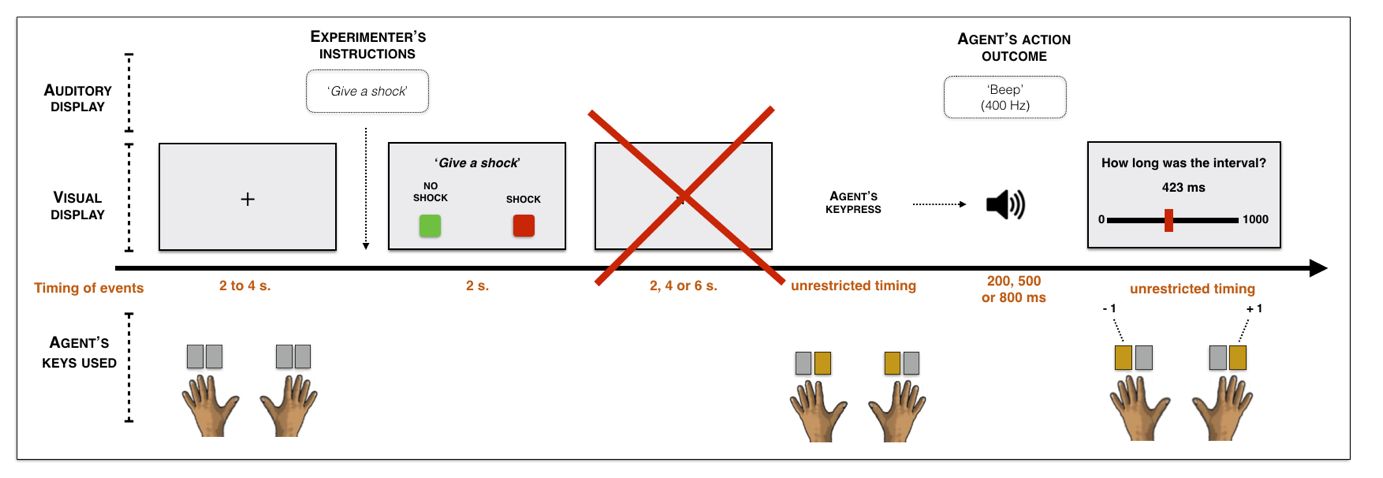


Fig. S2. Display of the agent’s task in pilot 2.

**PILOT 2. RESULTS**

***Interval estimates.*** We conducted a repeated-measures ANOVA with Condition (Free-choice, Coercive) and Shock (Shock, No shock) as within-subject factors on agent’s interval estimates. Three participants never inflicted shocks to the ‘victim’ in the free-choice condition and one inflicted shocks on every trial. These four participants were thus not taken into account in the ANOVA. The main effect of Condition was significant (F(1,14)=7.023, *p* = .019, η^2^***_partial_*** = .334), with shorter interval estimates in the free-choice condition (454 ms, 95%IC: 409-500) than in the coercive condition (488 ms, 95%IC: 450-526). Neither the main effect of outcome (*p* > .6) nor the interaction (*p* > .1) were significant.

***Electrophysiological results.*** A paired sample t-test indicated that the amplitude of the auditory N1 was larger in the free-choice condition (-10.63 µv, SD=3.84) than in the coercive condition (-9.27 µv, SD=4.12, t(18)=-2.258, *p* = .037, Cohen’s d=.515).

**Supp Mat S2**

Histogram of the number of freely administered shocks for participants performing the task inside the MRI scanner.


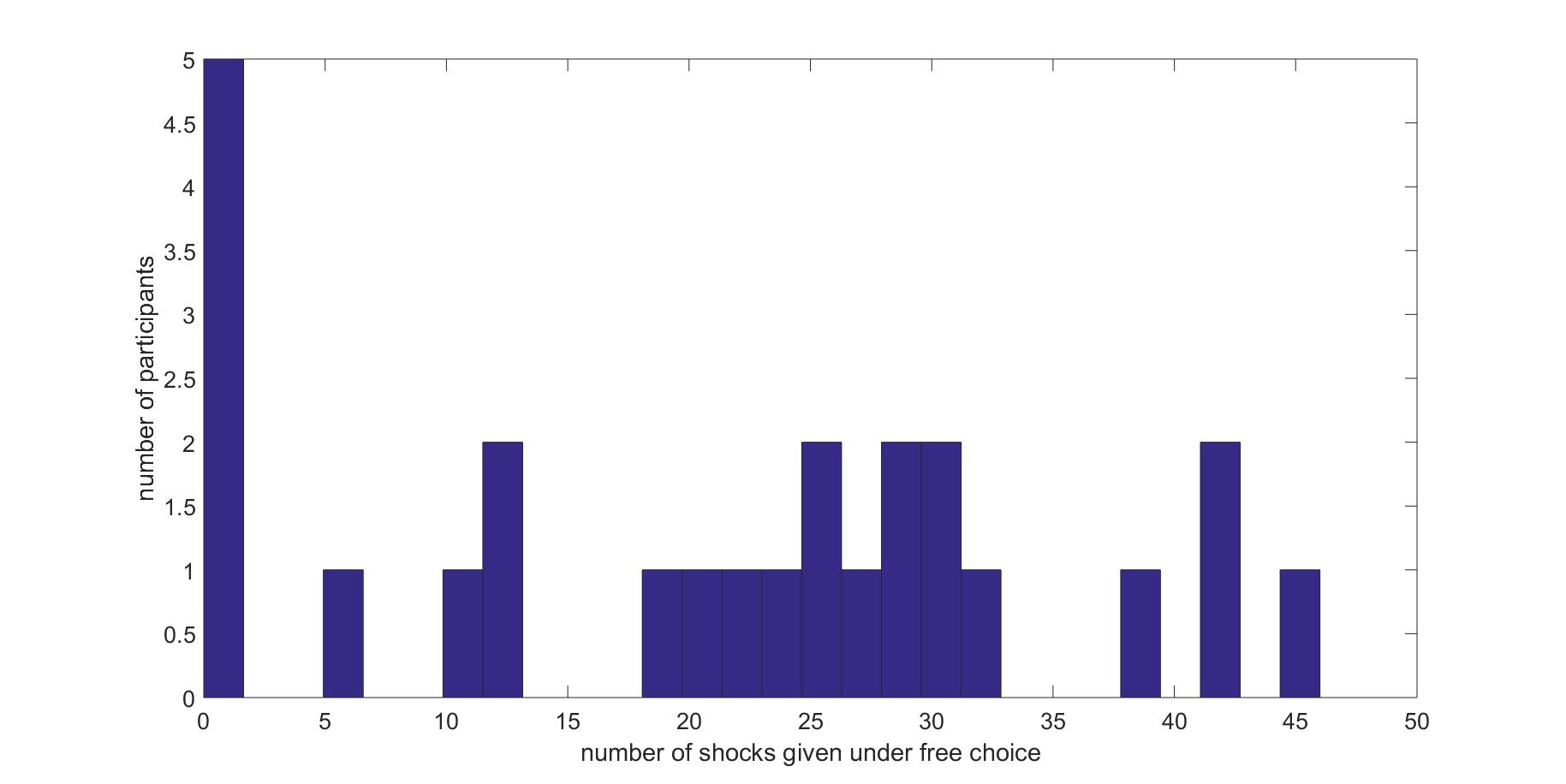


**Figure S2.1: number of shocks given under free choice.**

Results of regression analysis of number of freely administered shocks against free choice > coercion MRI contrast, including additional regressors of coercion effects on binding and responsibility ratings.

**
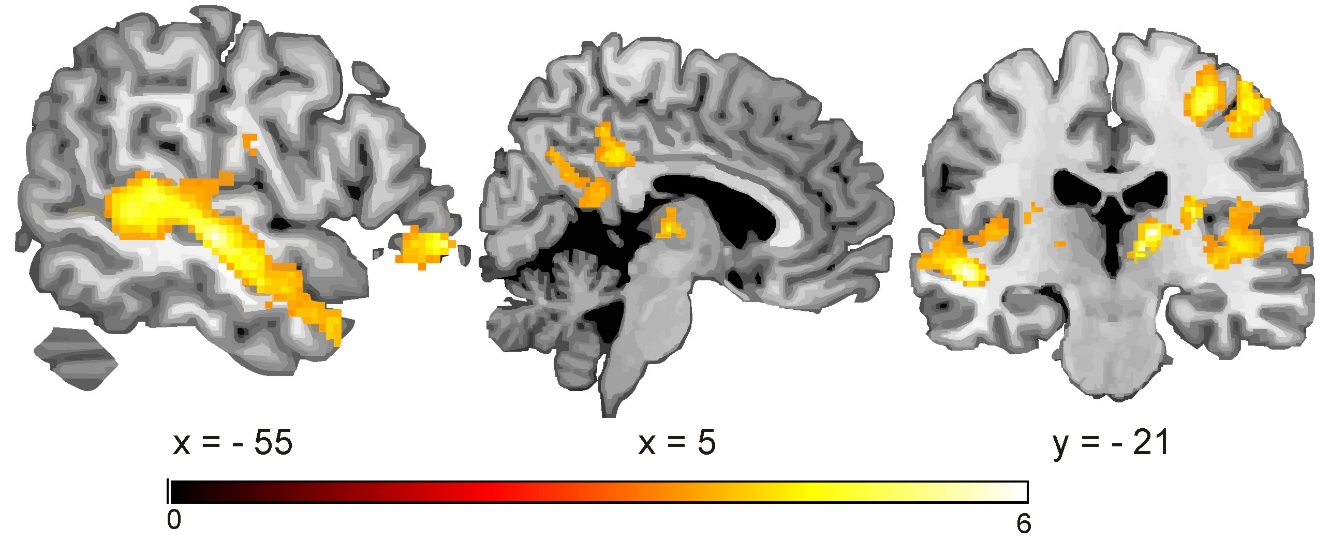
**

**Figure S2.2: regression of freely administered shocks against free choice > coercion contrast**. Significance threshold p < .001 uncorr., minimum cluster size = 100 voxels. For comparison, slices shown were chosen to correspond to those presented in the main article.

Caspar, E. A., Christensen, J. F., Cleeremans, A., & Haggard, P. (2016). Coercion Changes the Sense of Agency in the Human Brain. *Current Biology*, *0*(0).
